# Improved methods for mare milk analysis: Extraction and quantification of mare milk carbohydrates and assessment of FTIR-based macronutrient quantification

**DOI:** 10.3389/fnut.2023.1066463

**Published:** 2023-01-19

**Authors:** Morgan B. Pyles, Kristin Brock, Rachel R. Schendel, Laurie M. Lawrence

**Affiliations:** ^1^Department of Animal and Food Sciences, University of Kentucky, Lexington, KY, United States; ^2^Division of Regulatory Services, University of Kentucky, Lexington, KY, United States

**Keywords:** mare milk composition, milk oligosaccharides, Fourier transform infrared spectroscopy (FTIR), HPAEC-PAD, method validation, Kjeldahl protein analysis, Mojonnier fat analysis

## Abstract

Accurately determining the macronutrient profile of mare milk is a precursor to studying how milk composition affects foals’ growth and development. This study optimized and validated an extraction and quantification method for mare milk oligosaccharides, which make up a portion of the carbohydrate fraction of mare milk. Mare milk was extracted with chloroform and methanol, and oligosaccharides were selectively isolated from the carbohydrate fraction using porous-graphitized carbon solid-phase-extraction (SPE). Good recovery rates for milk oligosaccharides (between 70 and 100%) were achieved with the optimized method. This study also compared the use of Fourier-Transform infrared (FTIR) spectroscopy versus wet chemistry quantification methods for protein, fat, and lactose. The FTIR method produced statistically equivalent protein contents to the wet chemistry method, along with substantial savings in both analyst time and consumable consumption. FTIR analysis slightly underestimated the fat content of mare milk relative to the official wet chemistry method, with the difference between the methods increasing at higher fat contents. FTIR also overestimated the lactose content of mare milk and appeared to generate “lactose” values that included the milk oligosaccharides and thus represented the total carbohydrate (lactose and milk oligosaccharides) content of mare milk.

## 1. Introduction

Factors that affect the composition of mare milk have the potential to influence foal growth and development. Investigating the effects of different factors on milk composition in mares would be facilitated by the use of rapid methods to analyze milk components. The dairy industry has utilized infrared spectroscopy to analyze macronutrient composition of bovine milk for decades (1). Because of the availability, ease of use and rapid results, some researchers have used infrared analysis to evaluate mare milk composition (2–4). However, the method has yet to be validated for use in mare milk, which tends to be lower in fat and higher in lactose compared to bovine milk.

Using infrared spectroscopy, lactose concentration is often calculated based on absorption of light at characteristic frequencies in the spectral region between 1000 and 1200 cm^–1^. However, milk may also contain additional carbohydrates, such as milk oligosaccharides (MOS). In human medicine, it is now widely recognized that oligosaccharides in maternal milk have many beneficial functions including prebiotic, antibiotic, and immunomodulatory effects (5–8).

Milk oligosaccharides contain either lactose (Gal(β1-4)Glc) or N-acetyllactosamine (Gal(β1-4)GlcNAc) at their reducing end, with additional monosaccharides (glucose, galactose, N-acetylglucosamine, N-acetylgalactosamine, fucose, and sialic acids) branching off from the non-reducing galactose. Over 50 unique oligosaccharides have been found in mare milk (9–12). Previous research in MOS has focused on generating oligosaccharide profiles and investigating individual oligosaccharides. Various approaches have been used to analyze oligosaccharides in mare milk, but reliable methods to quantify the total oligosaccharide content in mare milk are lacking. Given the structural similarities between lactose and MOS, we hypothesized that the lactose concentration reported in studies of mare milk using infrared spectroscopy is actually an estimate of total milk carbohydrates (lactose plus oligosaccharides) rather than pure lactose. High-performance anion-exchange chromatography with pulsed amperometric detection (HPAEC-PAD) easily separates lactose from MOS, which enables the quantification of pure lactose. Under alkaline conditions, carbohydrates such as lactose and MOS are transformed into oxyanions and are separated on quaternary-ammonium-derivatized anion-exchange columns. Detection is accomplished electrochemically with a quadruple-potential pulsed waveform, which alternates positive and negative potential pulses at a gold working electrode surface to oxidize carbohydrates and renew the electrode surface (13, 14).

The objectives of this study were to (1) validate a method to quantify total oligosaccharides in mare milk and (2) assess the accuracy of Fourier transform infrared spectroscopy (FTIR) when measuring macronutrient composition of mare milk compared to reference methods.

## 2. Materials and methods

### 2.1. Animals and milk collection

All procedures were approved by the Institutional Animal Care and Use Committee at the University of Kentucky. Sixteen Thoroughbred mares were used for this study with a mean age of 13 yr (range 5–19 yr). The mares were fed a custom mixed oat and soybean hull-based pelleted concentrate three times daily. Mixed grass and alfalfa hay and cool-season grass pastures were available *ad libitum* throughout the study.

Milk samples were collected from mares at 12 h, 3, 7, 14, and 21 d postpartum. During sample collection, mare and foal pairs were brought into individual box stalls (3.6 × 3.6 m). The foals remained with their dam during sample collection. To ensure accurate representation of milk composition, colostrum/milk samples were collected by hand during natural milk let down while the foals were nursing. Milk was expressed into sterile specimen cups, aliquoted, and then frozen (−80°C) until analyzed. Milk samples were only exposed to one freeze-thaw cycle.

### 2.2. Extraction and quantification of milk carbohydrates

Total carbohydrates, lactose, and MOS were analyzed in 12 h, 3 d, and 7 d milk samples. The total carbohydrate content of all mare milk samples was quantified gravimetrically after extraction. Lactose was analyzed in all samples using FTIR spectroscopy (described below) and also *via* HPAEC-PAD. The total MOS method developed and validated in this study was adapted from Difilippo et al. (9). Total MOS were analyzed in duplicate.

### 2.3. Extraction of total milk carbohydrates

Total carbohydrates were extracted from milk samples using a chloroform/methanol extraction. Milk (5 mL) was combined with 20 mL of chloroform. After 2 h of head-over-tail mixing, the samples were centrifuged at 1000 × *g* for 30 min at 4°C and the aqueous phase was collected. The aqueous phase was treated with 2 volumes of methanol, vortexed and centrifuged at 2500 × *g* for 30 min at 4°C. The supernatant was transferred to pre-weighed tubes and dried *via* centrifugal evaporation. Once dried to a steady weight, the tubes containing extracted milk carbohydrates were re-weighed and the total carbohydrate content was calculated.

### 2.4. Lactose quantification with HPAEC-PAD

The lactose content was analyzed after carbohydrate extraction. The dried milk carbohydrates were solubilized in nanopure water to a concentration of 4 g/L, diluted 1:500 with water, and analyzed *via* HPAEC-PAD. The HPAEC-PAD analyses were performed on a Dionex ICS 5000+ DC series system equipped with an electrochemical detector (Thermo Fisher Scientific Inc., MA, USA). Milk carbohydrates were separated on a CarboPac PA-200 column (250 × 3 mm; 5.5 μm particle size) connected to a CarboPac PA-200 guard column (50 × 3 mm; 5.5 μm particle size). Elution was carried out using the following eluents and gradient: pure water (eluent A), 100 μM NaOH (eluent B) and 100 μM NaOH + 0.2 M NaOAc (eluent C); 0–20 min hold at 70% A, 30% B; 20–34 min, ramp B from 30 to 100%; 34–77 min, ramp C from 0 to 100%; 77–87 min hold 100% C to wash column; 87–107 min re-equilibrate column at 70% A, 30% B. The lactose peak was integrated using Dionex Chromeleon software (Thermo Fisher Scientific Inc., MA, USA) and lactose concentration was calculated using a 6-point standard curve (1–25 μM lactose).

### 2.5. Milk oligosaccharide clean-up and quantification

Solid phase extraction (SPE) was used to isolate the MOS by removing the majority of lactose. Dried milk carbohydrates were solubilized in nanopure water to a concentration of 50 g/L. SPE was performed using graphitized non-porous carbon cartridges (2 g bed weight, 12 mL volume, Supelclean Envi-carb, Sigma-Aldrich). Before samples were loaded, the SPE cartridges were washed with 1 tube volume (12 mL) of acetonitrile and conditioned with 2 tube volumes of nanopure water. Samples (100 mg of carbohydrates) were loaded onto the SPE cartridges, then salts were eluted with 2 tube volumes of water. Three tube volumes of 2% (v/v) acetonitrile/water solution were used to elute monomers and lactose from the cartridges. Then MOS were eluted in one tube volume of 40/60 (v/v) acetonitrile/water solution containing 0.05% (v/v) trifluoroacetic acid. The obtained oligosaccharides were dried to a constant weight in a centrifugal evaporator.

The amount of residual lactose in the MOS samples following SPE was quantified to adjust total MOS. The dried MOS were solubilized in nanopure water to a concentration of 4 g/L then diluted 1:500 with water. HPAEC-PAD was used to quantify the lactose content in post-SPE milk samples as described above.

### 2.6. Validation of oligosaccharide method

The method for extracting, purifying, and quantifying mare MOS was validated by evaluating both the ability of the SPE method to selectively reduce lactose and the recovery of oligosaccharides after SPE. To test the ability of the SPE method to fractionate lactose and MOS, lactose was quantified pre- and post-SPE. Milk carbohydrates were extracted from 3 mare milk samples at 7 d postpartum using the method previously described. The dried carbohydrates were resolubilized in water and injected on the HPAEC-PAD to quantify lactose pre-SPE. Then 100 mg of total milk carbohydrates were applied to SPE using the same protocol described above. After SPE, the total oligosaccharide solution was applied to HPAEC-PAD. Lactose was quantified in the pre- and post-SPE samples using a 6-point standard curve (1–25 μM lactose).

To test the recovery of MOS after SPE, the concentration-based response of mare MOS was evaluated. Stock solutions (4 g/L) were prepared in triplicate using total mare milk carbohydrates obtained following chloroform/methanol extraction but prior to SPE. The recovery procedure was repeated 3 times. A 6-point dilution series from 0.01 to 0.25% of mare milk carbohydrates was created from each stock solution. Each point was injected on the HPAEC-PAD, peak areas were recorded for 4 selected oligosaccharide peaks, and a calibration curve was created for each of the 4 peaks using linear regression. To evaluate recovery of oligosaccharides after SPE, 0.625 mL of each stock solution (2.5 mg of total carbohydrates) were loaded on to SPE cartridges (250 mg bed wt, 6 mL volume, Supelclean Envi-carb, Sigma-Aldrich, Inc. MO, USA) and total oligosaccharides were eluted following the same SPE protocol as described above. The final eluate containing oligosaccharides was analyzed on the HPAEC-PAD. The actual concentrations were calculated using the calibration curves and compared to the theoretical concentration. The percent recovery was calculated as follows:


$$\%\text{recovery}=\frac{A\,c\,t\,u\,a\,l\,c\,o\,n\,c\,e\,n\,t\,r\,a\,t\,i\,o\,n}{T\,h\,e\,o\,r\,e\,t\,i\,c\,a\,l\,c\,o\,n\,c\,e\,n\,t\,r\,a\,t\,i\,o\,n}\times 100$$

### 2.7. Protein and fat analysis using Fourier transform infrared spectroscopy and official reference methods

Fourier transform infrared spectroscopy spectroscopy on a specialized milk analysis instrument (MilkoScan™ FT+, Foss, Denmark) was used to analyze the concentrations of fat (*n* = 13), protein (*n* = 19), and lactose (*n* = 32) in mare milk samples. The FTIR analysis was conducted at the University of Kentucky Regulatory Services Milk Laboratory (Lexington, KY, USA). Mare milk samples were analyzed in duplicate. Milk samples were warmed in a water bath (40°C), then inverted several times to mix well before being presented to the FTIR analyzer pipette. The instrument was calibrated using raw lowfat non-fortified bovine milk (Eurofins DQCI, MN, USA). The composition of the bovine milk standards (Table 1) was verified at Eurofins DQCI using official reference methods (15): total solids content *via* forced air oven drying (AOAC 990.20), ash *via* muffle furnace (AOAC 945.46), protein content *via* Kjeldahl (AOAC 991.20), and fat content *via* Mojonnier (AOAC 989.05). Lactose content of the bovine milk standards was calculated by difference: % lactose = total solids% - fat% - total protein% - ash%. The protein of mare milk samples were determined by an official reference method (AOAC 991.20) using a Kjeltec™ 8400 Analyzer Unit (Foss, Denmark), and fat content of mare milk samples was determined using the same the same Mojonnier official reference method as described for the bovine milk standards. The total carbohydrate content of the mare milk samples was analyzed as described above. The mare milk lactose content was measured *via* HPAEC-PAD as described above.

**TABLE 1:** Composition and quality control of the FTIR low fat bovine milk standards^1^.

	Fat, %		Protein, %		Lactose, %	
**Standard**	**FTIR**	**Mojonnier**	**FTIR**	**Kjeldahl**	**FTIR**	**Calc by Diff^2^**
1	0.20	0.13	3.09	3.10	5.05	5.05
2	0.35	0.30	3.09	3.12	5.05	5.02
3	0.52	0.48	3.09	3.11	5.05	5.03
4	0.82	0.79	3.09	3.11	5.03	5.02
5	0.96	1.04	3.09	3.10	5.02	5.10
6	1.24	1.36	3.10	3.08	5.01	5.04
7	1.56	1.59	3.08	3.06	4.94	4.94
8	1.87	1.88	3.04	3.03	4.85	4.79
9	2.37	2.35	3.09	3.08	4.97	4.99
10	2.71	2.69	3.04	3.03	4.88	4.88
11	3.28	3.27	3.06	3.05	4.89	4.92
12	3.57	3.54	3.05	3.06	4.90	4.89
Standard dev		≤0.06 ≤0.06 ≤0.06				

^1^Results are the average of duplicate analysis.

^2^By diff lactose% = total solids% – fat% – total protein% – ash%.

### 2.8. Statistical analyses

The correlation between mare milk protein, fat, and lactose values from the FTIR method and reference methods were evaluated using regression analysis and Pearson’s correlation coefficient (Proc REG and CORR, SAS 9.4; SAS Institute Inc., NC, USA). Bland-Altman analyses were used to graphically represent the degree of bias (difference between each paired measurement) and the precision (± s.d. of the differences * 1.96 around the bias line) (16). Tests for zero bias with the FTIR and reference methods were analyzed using a paired *t*-test (SAS 9.4). Regression analysis (Proc REG, SAS 9.4) was used to create the calibration equations to quantify individual oligosaccharides when calculating recovery after SPE. Significance was considered when *P* < 0.05 and a trend considered when *P* < 0.10.

## 3. Results and discussion

### 3.1. Validation of the milk carbohydrate methods

Milk samples were collected from mares at 12 h, 3 d, 7 d, 14 d, and 21 d after parturition. The composition of mare milk changes with stage of lactation (2, 17). Colostrum is significantly higher in protein and lower in lactose compared to milk. Compared to other species, the transition from colostrum to milk occurs rapidly in mares, indicated by a significant decline in the protein content within the first 12 h after foaling (2). The samples collected in this study represent mare milk composition in early lactation, encompassing the transition from colostrum to milk (12 h and 3 d samples) and mature milk (7, 14, and 21 d samples).

Milk is a complex matrix containing all the nutrients needed to sustain the neonate. The complexity can create difficulties in evaluating specific nutrient groups within the milk, specifically the carbohydrates. To extract milk carbohydrates, fat and protein must first be removed. An extraction procedure utilizing chloroform and methanol to extract fat and protein, respectively, from mare milk has been used successfully in the isolation process for MOS (9, 12).

After fat and protein have been removed from the milk, the resulting solution will contain primarily lactose and oligosaccharides. Mare milk contains approximately 60–70 g/L lactose and 2–6 g/L total MOS (9, 11). A preparatory step is required to remove the more abundant lactose while retaining the MOS. The structural similarities between lactose and MOS can create challenges when purifying the MOS. Solid phase extraction has been used as a sample preparation step when analyzing MOS in bovine and porcine milk (18, 19), however, the method has not been validated for use with mare milk.

Three mare milk samples were used to assess the ability of the SPE method to separate lactose and oligosaccharides. The average lactose content in the milk samples before SPE was 57.12 g/L and was reduced to an average of 2.55 g/L after SPE. Thus, the SPE successfully removed approximately 96% of the lactose in the milk carbohydrate solution (Table 2). To evaluate the effectiveness of SPE for selective concentration of MOS with minimal losses, the recoveries of several oligosaccharide peaks before and after SPE were measured using HPAEC-PAD. There is limited availability for analytical standards for individual MOS. Thus, calibration curves were created for a subset of unidentified oligosaccharide peaks throughout the chromatogram (see Supplementary Figure 1). The HPAEC’s consistent separation pattern and retention times enabled reliable integration of the selected peaks between samples. Peak areas of the selected oligosaccharides were recorded and a calibration curve was created for each peak using dilutions of a mare milk carbohydrate stock solution. The relationship between the concentration and peak area was linear, with correlation coefficients between 0.974 and 0.994 (*P* < 0.0001; Table 3). In this study, the bed capacity of the SPE cartridges was 12.5 mg, 2.5 mg of total carbohydrates were applied to the cartridges and 70 to 99% of the selected oligosaccharides were recovered (Table 3). The high correlation coefficients and recoveries after SPE indicate that the method produces linear responses in peak area at different concentrations and there are minimal MOS losses during SPE cleanup.

**TABLE 2:** Separation of lactose and oligosaccharides in mare milk using solid phase extraction (SPE).

Lactose, mM	Pre-SPE	Post-SPE	% Lactose remaining
Sample A	6.938	0.385	5.55
Sample B	6.719	0.337	5.02
Sample C	6.855	0.355	5.18
Average	6.837	0.359	5.25

**TABLE 3:** Linearity of HPAEC-PAD^1^ response for selected milk oligosaccharides and recovery after solid phase extraction (SPE).

Peak	Regression equation^2^	Correlation coefficient (r)	% Recovery after SPE^3^			
			**Sample A**	**Sample B**	**Sample C**	**Avg**
1	Y = 4.439X+0.179	0.980	83.43	83.83	80.98	82.75
2	Y = 1.908X+0.001	0.994	96.57	99.81	85.50	93.96
3	Y = 1.034X−0.009	0.974	77.67	84.77	78.89	80.44
4	Y = 0.744X+0.013	0.974	80.97	81.58	70.06	77.54

^1^High performance anion exchange chromatography with pulsed amperometric detection.

^2^Y, peak area; X, nominal concentration (% of stock solution).

^3^Calculated using regression equations for peaks 1–4.

The affinity of lactose for the graphitized carbon solid phase used in this procedure is an important factor to consider when using SPE for MOS purification. Lactose uses a portion of the bed capacity on the solid phase during the sample loading step, thereby inhibiting the MOS from adhering if the cartridges are overloaded. Lactose and oligosaccharides are similar in structure (8), and thus both possess binding affinity to the graphitized carbon. However, elution with a 2% acetonitrile solution selectively removed the majority (96%) of the lactose. This method was successful in removing most of the lactose from the samples, however, quantifying the residual lactose in the final eluate is still necessary to accurately quantify the total MOS.

The recoveries of oligosaccharides after SPE in this study are higher than previous reports. Robinson et al. (20) measured recoveries of two individual oligosaccharides in bovine milk after SPE. The authors report an average recovery of 20.7 and 19.5% for 3′sialyllactose and 6′sialyllactose, respectively. In contrast, the recoveries of oligosaccharides after SPE in this study ranged from 70 to 99%. When evaluating the efficacy of SPE, the bed capacity of the cartridges used is an important factor to consider. Overloading the cartridge will result in loss of MOS. The typical manufacturer recommendation for bed capacity of SPE cartridges is 5% of the bed weight. In the study by Robinson et al. (20), the bed capacity was 2 mg. The authors report that the samples applied to SPE contained 0.04 mg of oligosaccharides, much lower than the bed capacity, however, the samples also contained 14.3 mg of lactose. Thus, the total carbohydrates applied to the SPE cartridges exceeded the bed capacity, contributing to the low recoveries of oligosaccharides. The results in the current study demonstrate that the SPE method used effectively removed the majority of the lactose with minimal losses of MOS.

An alternative option to remove lactose from the milk samples could include enzymatic digestion of lactose in combination with SPE. This combination has been successfully used to isolate MOS from human milk (21). The use of enzymatic digestion may be useful for isolating oligosaccharides in large quantities of milk and for further investigation into the structure and function of these molecules.

### 3.2. Comparison of FTIR and reference methods for mare milk components

Fourier transform infrared spectroscopy exposes the milk sample to radiation in the mid-infrared region. Different covalent bonds adsorb separate wavelengths of infrared radiation, which permits simultaneous quantification of the milk macronutrients (protein, fat, and lactose) *via* the amide groups in proteins, C-H bonds from the fatty acids, and C-H bonds in lactose. The concentration of the macronutrients is proportional to the amount of energy absorbed at designated specific wavelengths for each macronutrient. Additionally, energy adsorption is dependent on the product matrix (i.e., bovine milk vs mare milk). Thus, unique calibration coefficients are used to calculate the nutrient concentration for each type of product and are stored in a file on the instrument. Researchers have commonly used infrared analysis for mare milk due to the rapid results and ease of use (2, 22). However, the method has yet to be validated for use in mare milk against official laboratory methods.

When using FTIR, the milk sample is tested against the calibration curve created from the set of standards. The instrument used in this study is typically calibrated for bovine milk. The composition of mare milk is quite different from bovine milk; the fat content is lower and lactose is higher in mare milk compared to bovine milk. Thus, it was necessary to adjust the calibration curve to reflect the typical composition of mare milk rather than bovine milk. The standards for the FTIR instrument were selected based on the fat content expected in the mare milk samples. The low fat standards used in this study ranged in fat content from 0.20 to 3.57% (Table 1) which encompassed the range of milk fat in the mare milk samples (0.93 to 2.92 %).

To validate the use of FTIR for mare milk, a set of samples was analyzed both *via* official reference methods and FTIR analysis. Milk protein values from the FTIR were compared to values produced by the Kjeldahl method. Milk fat values were compared to the Mojonnier method, and milk lactose was compared to total carbohydrates, measured gravimetrically, and to lactose, measured using HPAEC-PAD. The correlation coefficients for protein and fat were >0.99, indicating a strong correlation between the values produced by the FTIR instrument and the reference methods. The correlation coefficient was 0.98 for total carbohydrates and 0.93 for HPAEC lactose (see Supplementary Figures 2, 3). The correlation coefficient provides a measure of association but is not useful for determining if the two methods are comparable. Regression analysis and Bland-Altman plots can be used to compare two methods.

**FIGURE 1:**
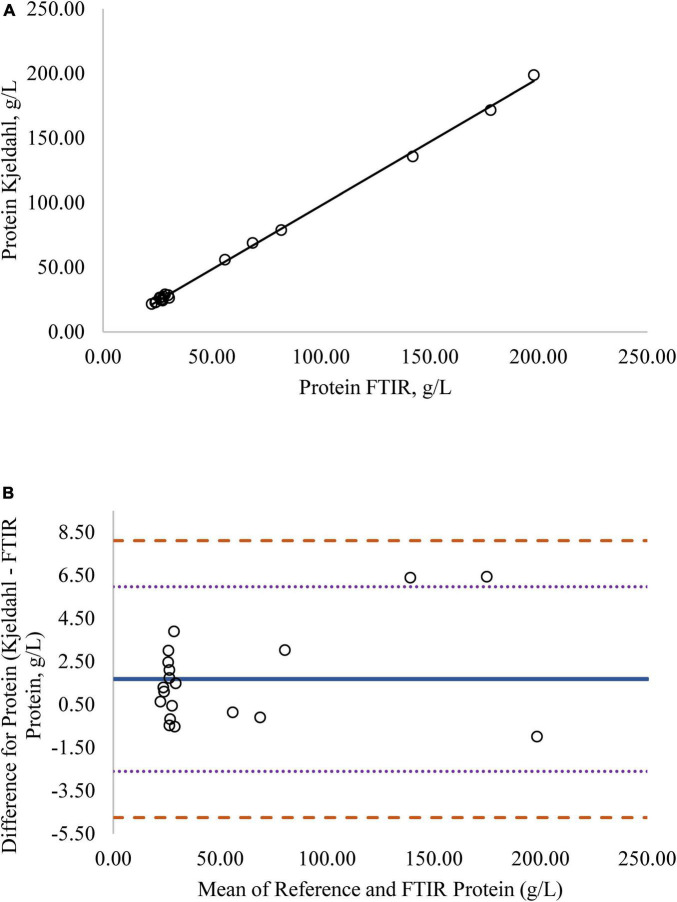
**(A)** Mare milk protein concentration measured by Fourier transform infrared spectroscopy (FTIR) and the reference Kjeldahl method. Correlation graph between the two methods for protein, *r*^2^ = 0.99, *P* < 0.0001. Regression model y = 0.986X – 0.088. **(B)** Mare milk protein concentration measured by FTIR and Kjeldahl method. Bland-Altman plot showing the mean differences and limits of agreement. Bias line: solid line, two standard deviation (s.d.): dotted lines, three s.d.: dashed lines.

**FIGURE 2:**
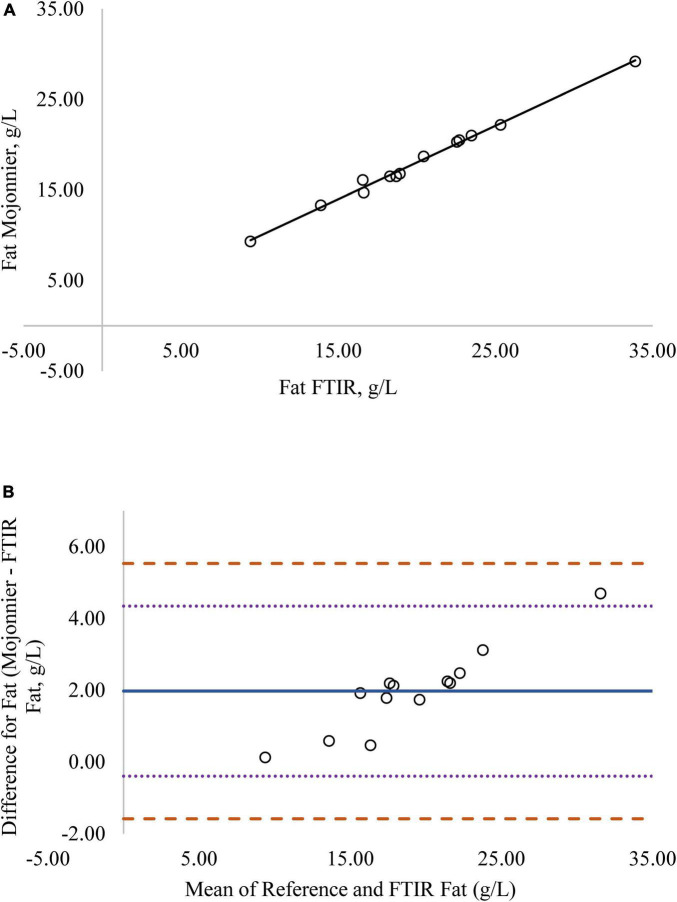
**(A)** Mare milk fat concentration measured by FTIR and the reference Mojonnier method. Correlation graph between the two methods for fat, *r*^2^ = 0.99, *P* < 0.0001. Regression model y = 0.812X + 0.180. **(B)** Mare milk fat concentration measured by FTIR and Mojonnier method. Bland-Altman plot showing the mean differences and limits of agreement. Bias line: solid line, two standard deviation (s.d.): dotted lines, three s.d.: dashed lines.

**FIGURE 3:**
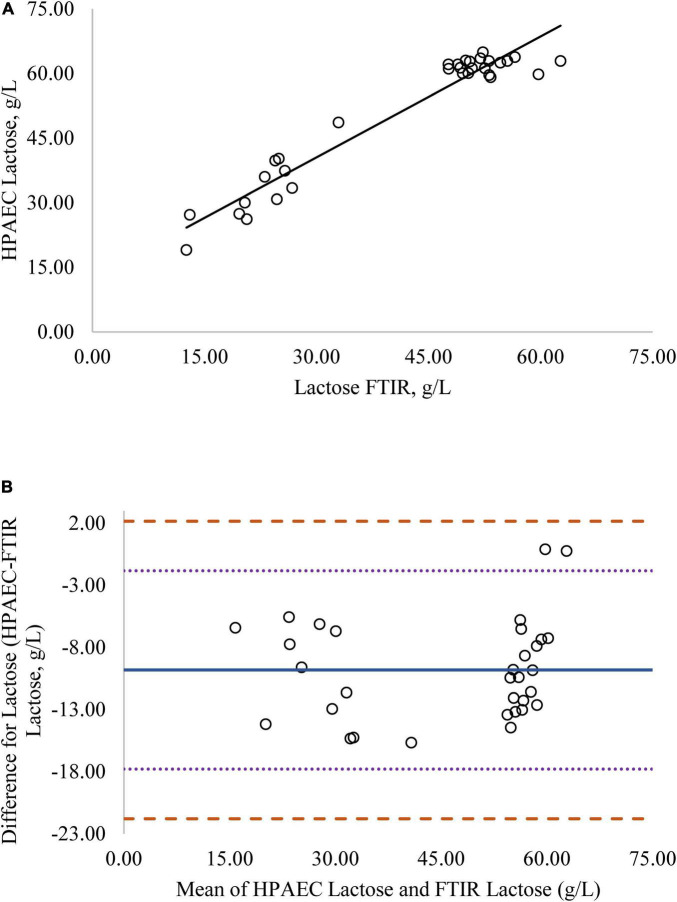
**(A)** Mare milk lactose concentration measured by FTIR and High Performance Anion Exchange Chromatography (HPAEC) method. Correlation graph between the two methods for lactose, *r*^2^ = 0.93, *P* < 0.0001. Regression model y = 0.950X + 11.837. **(B)** Mare milk lactose concentration measured by FTIR and HPAEC method. Bland-Altman plot showing the mean differences and limits of agreement. Bias line: solid line, two standard deviation (s.d.): dotted lines, three s.d.: dashed lines.

**FIGURE 4:**
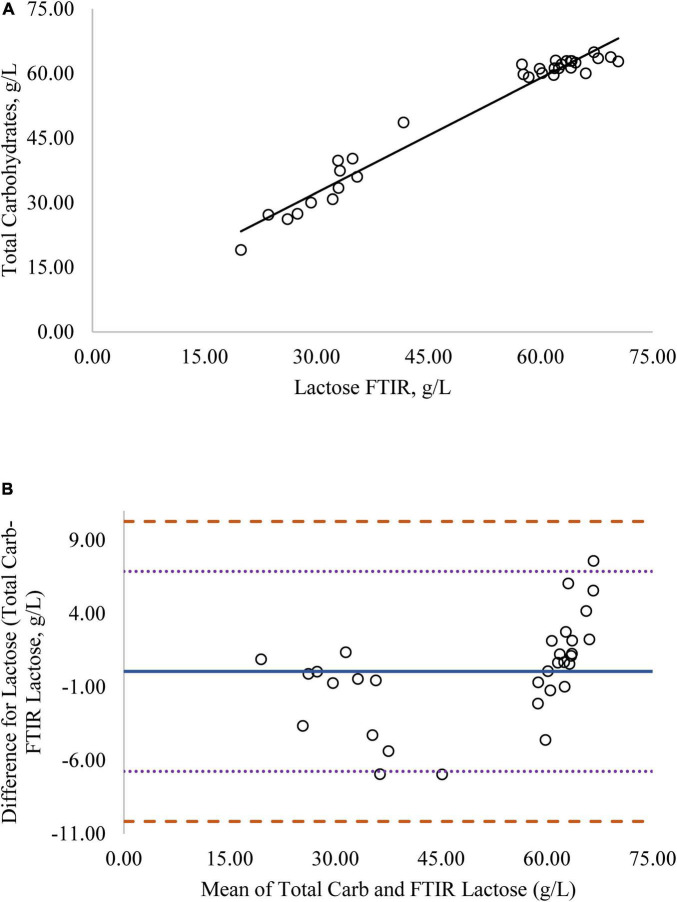
**(A)** Mare milk lactose concentration measured by FTIR and total carbohydrate method. Correlation graph between the two methods for lactose, *r*^2^ = 0.98, *P* < 0.0001. Regression model y = 0.920X + 4.512. **(B)** Mare milk lactose concentration measured by FTIR and total carbohydrate method. Bland-Altman plot showing the mean differences and limits of agreement. Bias line: solid line, two standard deviation (s.d.): dotted lines, three s.d.: dashed lines.

Regression analysis was used in the comparison of FTIR-determined protein, fat, and lactose against the reference methods and revealed that the reference methods were linearly related to the FTIR values (Figures 1A–4A, respectively; *P* < 0.0001). If the methods are perfectly equivalent, then the slope of the regression line should be 1. The slopes of the regression equations for protein, HPAEC lactose, and total carbohydrates were not different from 1 (*P* > 0.05; Figures 1, 3, 4, respectively). The slope of the regression for fat was 0.812 and was different from 1 (*P* < 0.05; Figure 2A), indicating that the FTIR may underestimate milk fat as the concentration increases.

Bland-Altman plots for protein, fat, and lactose (Figures 1B–4B) visualize the differences between the methods. The two standard deviation (s.d.) limits (dotted lines) estimate where 95% of the differences should be, and 99% of the differences should be within three s.d. (dashed lines). The bias line (solid line) aids in determining if one method produces values higher or lower than the other, on average. The degree of bias was 1.68 for protein, 1.98 for fat, 0.059 for total carbohydrates, and −9.81 for HPAEC lactose. The test for zero bias for total carbohydrates compared to FTIR was not different from 0 (bias = 0.059; *P* = 0.922), thus the FTIR produces values similar to total carbohydrates. However, HPAEC lactose was significantly lower than FTIR lactose (bias of −9.811; *P* < 0.001) indicating that FTIR overestimated true lactose in this study. The bias test for protein and fat indicated that, on average, the FTIR produces values lower than the reference methods (*P* < 0.05). The differences between the methods were distributed relatively equally above and below the bias line for protein, total carbohydrates, and lactose. However, for fat the difference between the two methods increased as the concentration of fat increased.

This study assessed the accuracy of FTIR for the analysis of the macronutrient composition of mare milk by comparing the FTIR method to standard laboratory methods. Standard laboratory methods are expensive due to the chemical requirements, need additional equipment, and are significantly more time-consuming compared to infrared spectroscopy. The FTIR method does not require chemicals and is a rapid test. Although many studies have used infrared analysis to determine the composition of mare milk (2–4, 23), no studies have validated infrared analysis for use with mare milk. Previous research has validated the use of infrared analyzers for human milk by comparing the method to standard laboratory methods (24–26). The agreement between infrared analysis and reference methods were relatively high for protein and fat and the actual differences between the two methods were relatively small numerically.

Although the correlation between FTIR lactose and HPAEC lactose was high, the numerical difference in the values produced by the two methods was significant, as indicated by the lack of zero bias and intercept (Figure 3). Although lactose is the primary carbohydrate in mare milk, mare milk also contains over 50 oligosaccharides (9–11). The structures of MOS are diverse but most contain a core structure of a lactose unit at the terminal end (27). Thus, both lactose and oligosaccharides will be detected when subjected to FTIR. The large difference in values for lactose from the FTIR to HPAEC lactose could be explained by the presence of oligosaccharides in mare milk. The HPAEC analysis chromatographically separates lactose and oligosaccharides and produces values for true lactose content. There was zero bias and higher correlation between FTIR lactose and total carbohydrates measured in mare milk (Figure 4). These results indicate that the values for lactose produced by the FTIR instrument appear to represent the total carbohydrate content, which includes both lactose and oligosaccharides, rather than lactose alone.

Differences in MOS concentration has been observed in women in different geographical locations (28). Differences were observed between women despite expected genetic similarity. These results led the authors to believe that environmental factors, such as diet, may have led to the differences in MOS. Furthermore, a recent study investigated the effect of diet on MOS in women (29). The authors reported that women consuming a diet rich in carbohydrates produced higher concentrations of sialic acid-containing MOS compared women consuming an isoenergic high fat diet. The role of genetic background, geographical location, and diet on mare milk composition, particularly oligosaccharide content, has received little attention. The methods developed and validated in the current study will enable investigation of this important topic that has implications to foal health.

## 4. Conclusion

This study aimed to validate a method for quantification of total MOS in mare milk and to evaluate the use of FTIR to analyze the macronutrient composition of mare milk. The results suggest that FTIR measurement of protein and fat may yield acceptable results in mare milk. However, the resulting values for lactose produced by the FTIR appear to represent the total carbohydrate content, including both lactose and oligosaccharides. This study demonstrated that the use of SPE as a preparatory step in quantifying oligosaccharides in mare milk is effective in the removal of lactose with minimal losses of oligosaccharides.

## Data availability statement

The raw data supporting the conclusions of this article will be made available by the authors, without undue reservation.

## Ethics statement

The animal study was reviewed and approved by the Institutional Animal Care and Use Committee at the University of Kentucky.

## Author contributions

MP contributed to the conception of the study and experimental design (along with RS and LL), sample and data analysis, data interpretation, and drafting of the manuscript. KB contributed to the sample analysis by performing Kjeldahl, Mojonnier, and FTIR analysis. RS substantial contribution to the method validation, HPAEC-PAD and SPE analysis, and drafting the manuscript. LL substantial contribution to conception of the study and experimental design (together with MP), data interpretation, and drafting of the manuscript. All authors have critically revised the manuscript for important intellectual content and approved the publication of its content.
