# Supplementary material for: Improved methods for mare milk analysis: Extraction and quantification of mare milk carbohydrates and assessment of FTIR-based macronutrient quantification
Source: Front Nutr. 2023 Jan 19;10:1066463. doi: 10.3389/fnut.2023.1066463 (PMC9892553; doi:10.3389/fnut.2023.1066463)
Supplement: Supplementary file 1 [file Data_Sheet_1.PDF]

Supplemental Figures

Article Title: Improved methods for mare milk analysis: Extraction and quantification of mare milk carbohydrates and assessment of FTIR-based macronutrient quantification

Authors: Morgan B. Pyles, Kristin Brock, Rachel R. Schendel, Laurie M. Lawrence

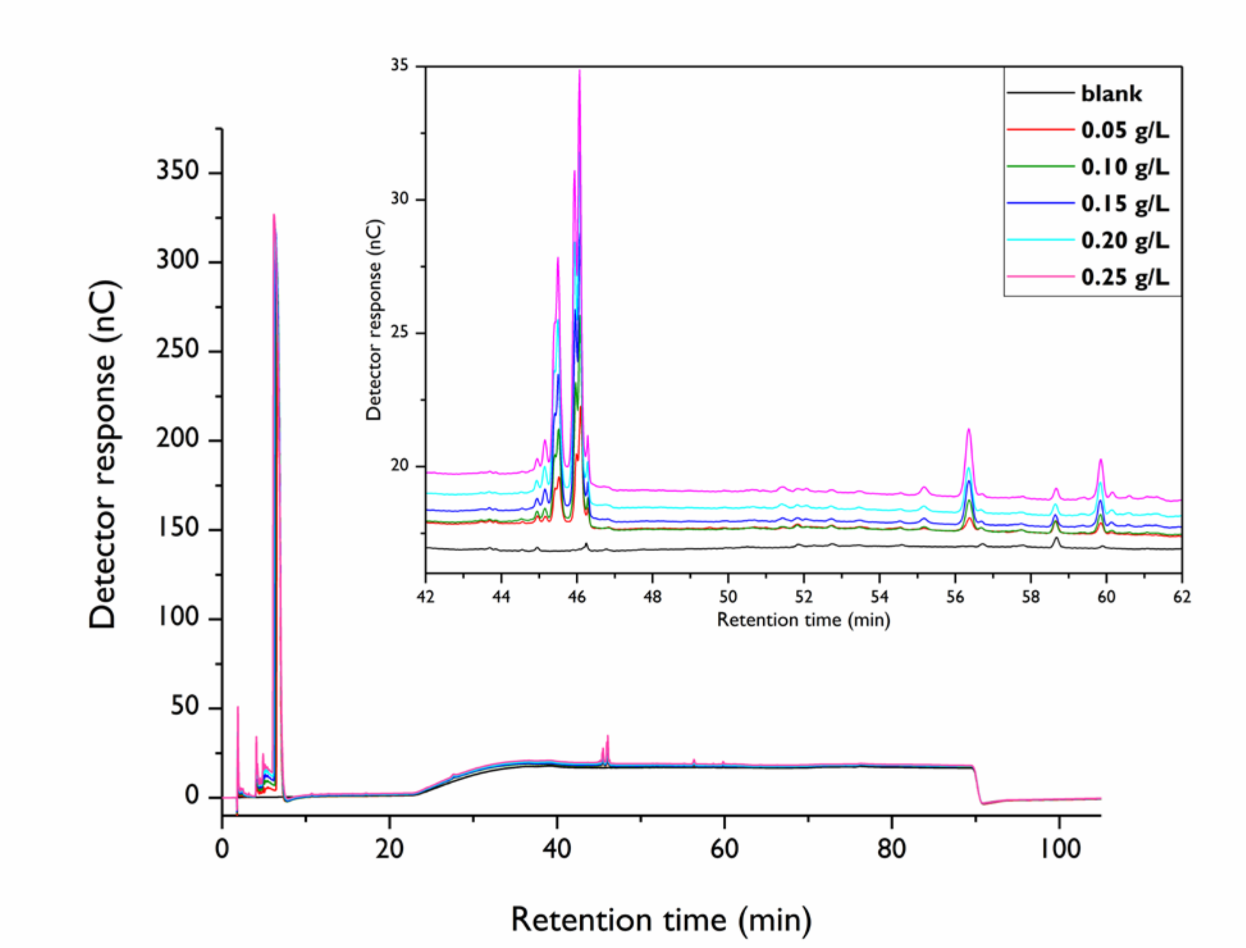

Supplemental Figure 1. HPAEC-PAD chromatograms corresponding to blank sample and serial dilutions of total milk carbohydrates prior to SPE clean-up (inset=zoomed-in view of oligosaccharide region). Peaks at 45.9, 46.05, 56.3, and 59.8 min retention time were integrated, and standard curves were created for each peak. Milk carbohydrates were then cleaned up using SPE, re-analyzed on the HPAEC-PAD, and the recovery of the milk oligosaccharides was assessed by quantification of the four selected peaks.

Abbreviations: HPAEC-PAD: high-performance anion-exchange chromatography coupled with pulsed amperometric detection; SPE: solid-solid phase extraction.

Supplemental Figures

Article Title: Improved methods for mare milk analysis: Extraction and quantification of mare milk carbohydrates and assessment of FTIR-based macronutrient quantification

Authors: Morgan B. Pyles, Kristin Brock, Rachel R. Schendel, Laurie M. Lawrence

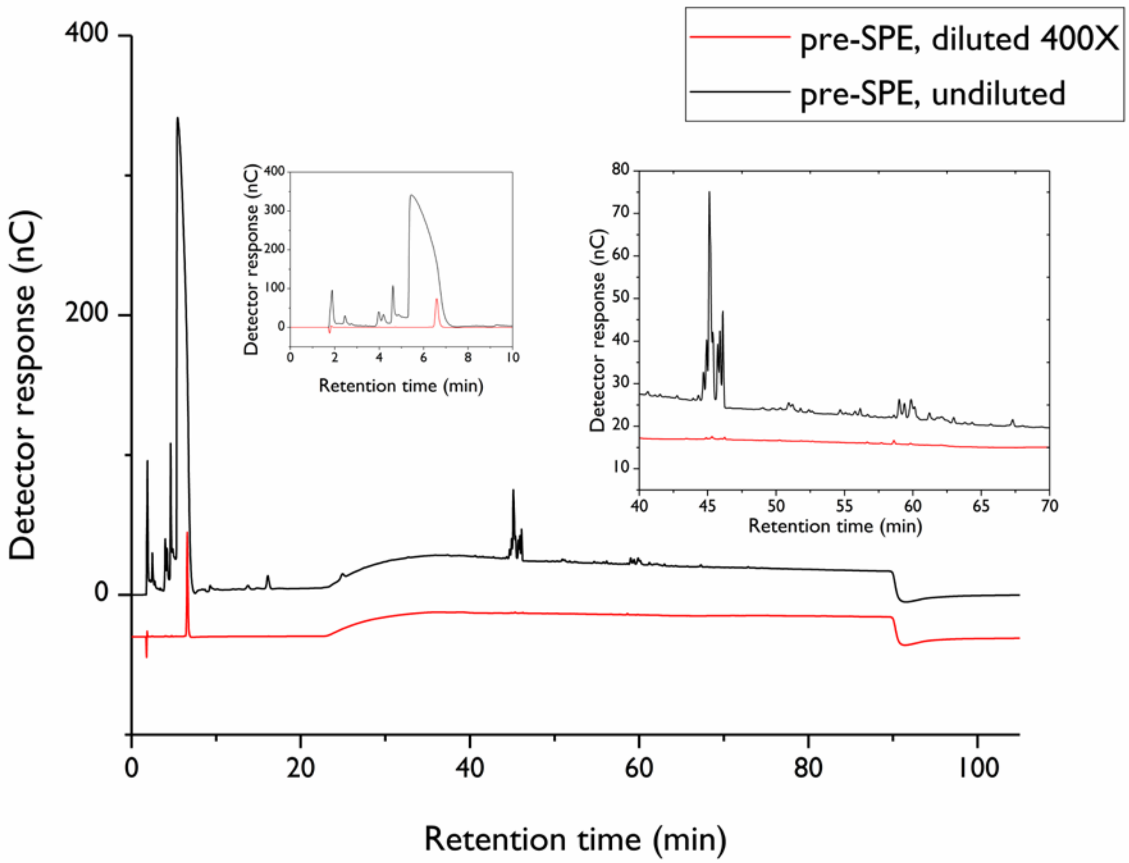

Supplemental Figure 2. Representative HPAEC-PAD chromatogram of total milk carbohydrates before and after dilution to quantify lactose. Lactose was quantified by diluting samples to bring lactose peak area into range of the lactose standard curve (see Supplemental Figure 3). Left inset: Zoomed-in view of lactose peak. Right inset: Zoomed-in view of oligosaccharide region.

Abbreviations: HPAEC-PAD: high-performance anion-exchange chromatography coupled with pulsed amperometric detection.

Supplemental Figures

Article Title: Improved methods for mare milk analysis: Extraction and quantification of mare milk carbohydrates and assessment of FTIR-based macronutrient quantification

Authors: Morgan B. Pyles, Kristin Brock, Rachel R. Schendel, Laurie M. Lawrence

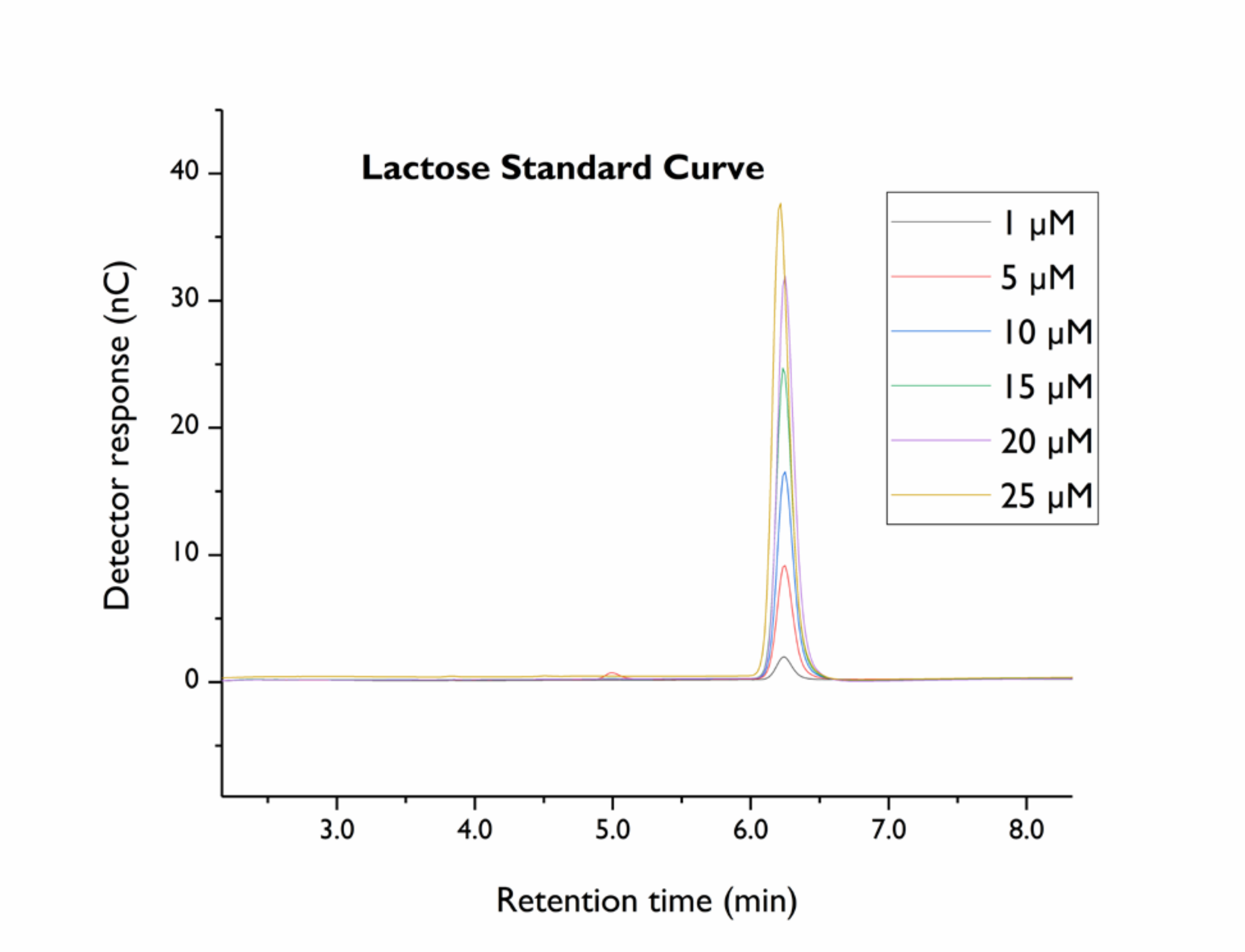

Supplemental Figure 3. Lactose peaks from representative standard curve analyzed via HPAEC-PAD. A new standard curve was prepared and analyzed with each batch of HPAEC eluent.

Abbreviations: HPAEC-PAD: high-performance anion-exchange chromatography coupled with pulsed amperometric detection.
